# Reproducibility of Aorta Segmentation on 4D Flow MRI in Healthy Volunteers

**DOI:** 10.1002/jmri.27431

**Published:** 2020-11-11

**Authors:** Joe F. Juffermans, Jos J.M. Westenberg, Pieter J. van den Boogaard, Arno A.W. Roest, Hans C. van Assen, Roel L.F. van der Palen, Hildo J. Lamb

**Affiliations:** ^1^ Department of Radiology Leiden University Medical Center Leiden The Netherlands; ^2^ Department of Paediatric Cardiology Leiden University Medical Center Leiden The Netherlands

**Keywords:** aorta, segmentation, aortic diameter, 4D flow MRI, reproducibility, repeatability

## Abstract

**Background:**

Hemodynamic aorta parameters can be derived from 4D flow MRI, but this requires lumen segmentation. In both commercially available and research 4D flow MRI software tools, lumen segmentation is mostly (semi‐)automatically performed and subsequently manually improved by an observer. Since the segmentation variability, together with 4D flow MRI data and image processing algorithms, will contribute to the reproducibility of patient‐specific flow properties, the observer's lumen segmentation reproducibility and repeatability needs to be assessed.

**Purpose:**

To determine the interexamination, interobserver reproducibility, and intraobserver repeatability of aortic lumen segmentation on 4D flow MRI.

**Study Type:**

Prospective and retrospective.

**Population:**

A healthy volunteer cohort of 10 subjects who underwent 4D flow MRI twice. Also, a clinical cohort of six subjects who underwent 4D flow MRI once.

**Field Strength/Sequence:**

3T; time‐resolved three‐directional and 3D velocity‐encoded sequence (4D flow MRI).

**Assessment:**

The thoracic aorta was segmented on the 4D flow MRI in five systolic phases. By positioning six planes perpendicular to a segmentation's centerline, the aorta was divided into five segments. The volume, surface area, centerline length, maximal diameter, and curvature radius were determined for each segment.

**Statistical Tests:**

To assess the reproducibility, the coefficient of variation (COV), Pearson correlation coefficient (*r*), and intraclass correlation coefficient (ICC) were calculated.

**Results:**

The interexamination and interobserver reproducibility and intraobserver repeatability were comparable for each parameter. For both cohorts there was very good reproducibility and repeatability for volume, surface area, and centerline length (COV = 10–32%, *r =* 0.54–0.95 and ICC = 0.65–0.99), excellent reproducibility and repeatability for maximal diameter (COV = 3–11%, *r =* 0.94–0.99, ICC = 0.94–0.99), and good reproducibility and repeatability for curvature radius (COV = 25–62%, *r =* 0.73–0.95, ICC = 0.84–0.97).

**Data Conclusion:**

This study demonstrated no major reproducibility and repeatability limitations for 4D flow MRI aortic lumen segmentation.

**Level of Evidence:**

3

**Technical Efficacy Stage:**

2

FOUR‐DIMENSIONAL (4D) flow magnetic resonance imaging (MRI), also known as time‐resolved three‐directional and three‐dimensional velocity‐encoded MRI or phase‐contrast MRI, is an imaging modality that is used to analyze aortic flow hemodynamics. With 4D flow MRI, multiple patient‐specific flow properties can be quantified, such as the wall shear stress.^1^, ^2^, ^3^ It has been hypothesized that changes in wall shear stress may affect endothelium properties within the vessel wall,^4^ which may promote vascular dilation and remodeling.^5^

For the numerical calculation of the 4D flow MRI‐derived hemodynamic parameters, a cardiac phase‐specific 3D lumen segmentation of the aorta is required.^3^, ^6^ In both commercially available and research 4D flow MRI software tools, lumen segmentation is mostly (semi‐)automatically performed and subsequently manually improved by an observer. Therefore, the observer's lumen interpretation may lead to segmentation variability.^2^, ^3^, ^7^, ^8^ The aortic lumen segmentation reproducibility was not assessed in previous studies that evaluated the reproducibility of several 4D flow MRI‐derived hemodynamic parameters.^8^, ^9^, ^10^, ^11^, ^12^ Since the segmentation variability, together with 4D flow MRI data and image processing algorithms, contributes to the reproducibility of patient‐specific flow properties,^6^ the observer's lumen segmentation reproducibility and repeatability needs to be assessed.

Furthermore, the aortic lumen segmentation could be used to automatically derive several morphological parameters, like the maximal vessel diameter. Clinical guidelines recommend measurement of maximal diameter perpendicular to the vessel longitudinal axis for the highest reproducibility.^13^, ^14^ This recommendation is challenging for observers, since they have to manually determine the optimal plane location and angulation towards the vessel.^15^ These difficulties could potentially be minimized by automatically deriving the maximal diameter from a lumen segmentation. However, as a result of the unknown lumen segmentation reproducibility, it remains uncertain if the automatically derived maximal diameter could be used to accurately describe patient characteristics. This information is especially important for the clinical follow‐up of patients with aorta pathologies, such as aneurysms or coarctations.^13^, ^14^

Therefore, the purpose of this study was to determine: 1) interexamination, 2) interobserver reproducibility, and 3) intraobserver repeatability of aortic lumen segmentation on 4D flow MRI.

## Materials and Methods

### *Study Population*

This study protocol was approved by the Medical Ethics Committee of the Leiden University Medical Center and written informed consent was obtained from all subjects. The interexamination reproducibility and intraobserver repeatability was assessed in a healthy volunteer cohort, and the interobserver reproducibility was assessed in a healthy volunteer cohort and a clinically relevant cohort. The prospective included a healthy cohort consisting of 10 healthy volunteers (27 ± 3 years, 50% male) without a history of cardiovascular disease who underwent two 4D flow MRI examinations between July 2015 and March 2017. These examinations were planned consecutively with a 10‐minute break between them and included repositioning and replanning of all subjects. The retrospective included a clinical cohort consisting of two patients after surgical coarctation repair (13 ± 1 years, one male and one female, one with a restenosis, and one with a bicuspid aortic valve), two aneurysm patients (65 ± 8 years, one male and one female), and two older healthy volunteers (59 ± 5 years, one male and one female) who underwent a 4D flow MRI examination between September 2015 and November 2019. The data from 10 of the 10 subjects of the healthy volunteer cohort has been previously reported^10^ in a prior article that assessed the interexamination, interobserver, and intraobserver reproducibility of 3D wall shear stress in the thoracic aorta.

#### *MRI ACQUISITION*

The MRI examination consisted of a 4D flow MRI sequence incorporating the thoracic aorta from the aortic valve to descending aorta at the level of the diaphragm. For the MRI sequence parameters, see Table 1. All subjects were scanned with a 3T scanner (Ingenia, Philips Medical Systems, Best, The Netherlands) using a FlexCoverage anterior and dStream Torso posterior coil. Concomitant gradient correction and local phase correction were performed using standard available scanner software.

**TABLE 1:** MRI Sequence Parameters

Parameter	4D Flow MRI
Respiratory compensation	Hemidiaphragm respiratory navigator
Electrocardiographic gating	Retrospective
Field of view [mm^3^]	350 × 250 × 75
Acquired spatial resolution [mm^3^]	2.5 × 2.5 × 2.5
Temporal resolution [msec]	35.1–36.5
Echo time [msec]	2.5–2.7
Repetition time [msec]	4.4–4.6
Flip angle [degree]	10
Planned acquisition time^a^ [seconds]	403 ± 35
Turbo field echo	2
Acceleration method	SENSE 2.5 in anterior–posterior direction
Velocity encoding gradient [cm/s]	200

Data notated as the mean ± standard deviation.

^a^ Excluding hemidiaphragm respiratory navigator.

#### *IMAGE ANALYSIS*

The image analysis consisted of two parts to derive the aorta morphology. First, the thoracic aorta lumen was segmented between the aortic valve and the descending aorta, excluding the subclavian and carotid arteries. The aortas of the healthy cohort were segmented twice by the first observer (R.P. with 6 years 4D flow MRI experience) on the first 4D flow MRI, once by the first observer on the second 4D flow MRI, and once by the second and third observers (P.B. and J.J. with 12 and 3 years 4D flow MRI experience, respectively) on the first 4D flow MRI. The aortas of the clinical cohort were segmented once by the first, second, and third observerd. The interobserver analysis between the first and second, first and third, and second and third observers are numbered 1, 2, and 3, respectively.

**FIGURE 1:**
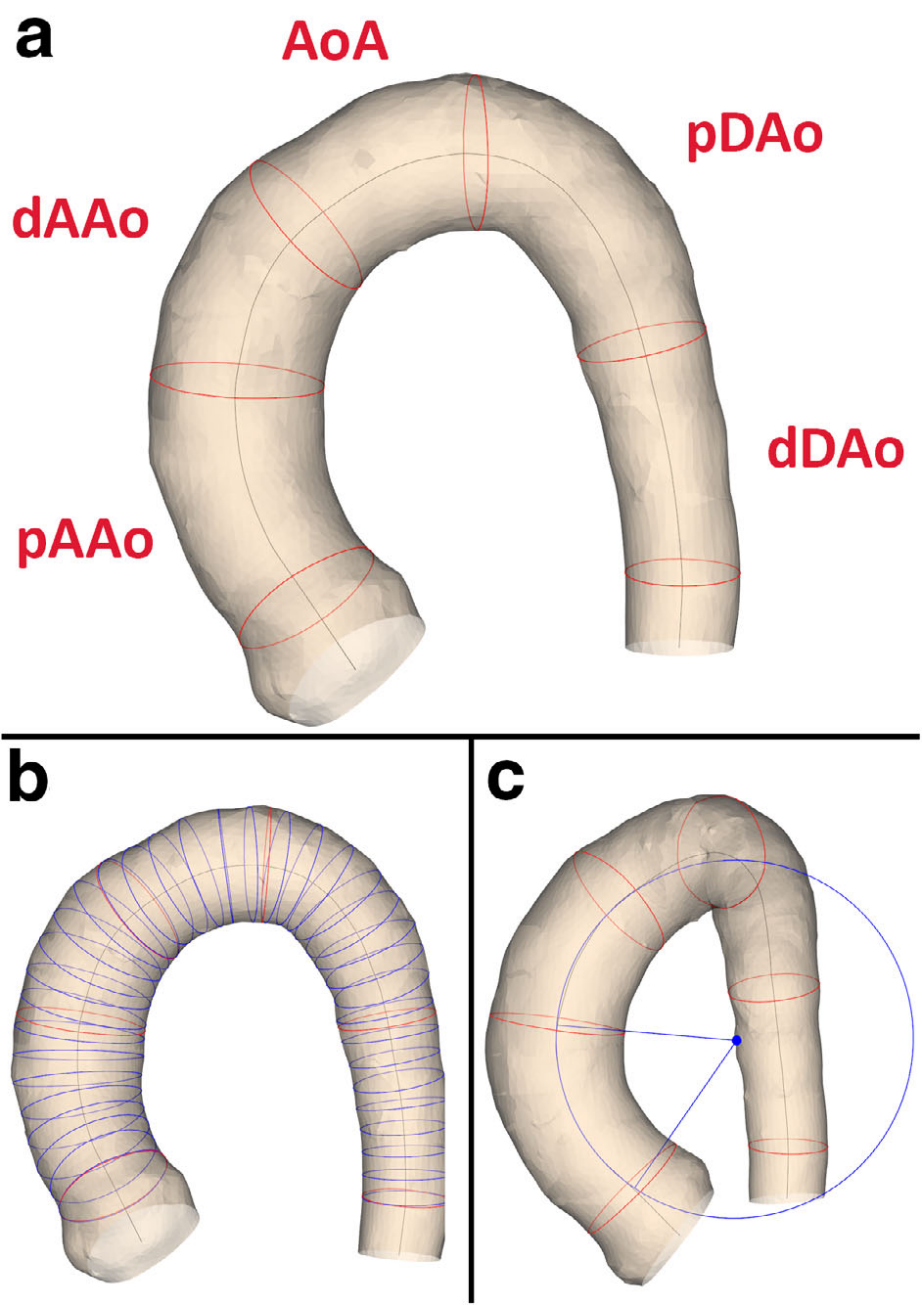
The aortic lumen segmentation with (**a**) the anatomical segments, (**b**) the lumen cross‐section to derive maximal diameter, (**c**) and a circle fitted to the proximal ascending aorta. Example of an aorta lumen segmentation of a healthy volunteer. (a) The anatomical segments. (b) The cross‐sections to derive the lumen diameter. To improve visibility, the cross‐sections are displayed every 5 mm instead of every mm that was used during the analysis. (c) A circle fitted to the proximal ascending aorta. pAAo = proximal ascending aorta (from the sinotubular junction to the mid‐ascending aorta), dAAo = distal ascending aorta (from the mid‐ascending aorta to the brachiocephalic artery), AoA = aortic arch (from the brachiocephalic artery up and including the left subclavian artery), pDAo = proximal descending aorta (from the left subclavian artery to the mid‐descending thoracic aorta) and dDAo = distal descending (from the mid‐descending thoracic aorta to the descending aorta at the level of the aortic valve).

The 4D flow segmentation was performed from a combined weighted magnitude and velocity image with CAAS MR 4D Flow v1.1 and CAAS MR Solutions v5.1 (Pie Medical Imaging, Maastricht, The Netherlands). Utilizing CAAS software, the peak systolic phase and two consecutive phases before and two after this peak systolic phase were segmented. By manually placing start and endpoints in the aorta, a lumen segmentation was automatically created, which subsequently was manually improved for each phase. Next, the thoracic aortic lumen was divided into five consecutive segments by manually placing anatomical planes perpendicular to the aortic centerline: the proximal ascending aorta (from the sinotubular junction to the mid‐ascending aorta), distal ascending aorta (from the mid‐ascending aorta to the brachiocephalic artery), aortic arch (from the brachiocephalic artery up and including the left subclavian artery), proximal descending aorta (from the left subclavian artery to the mid‐descending thoracic aorta), and distal descending aorta (from the mid‐descending thoracic aorta to the descending aorta at the level of the aortic valve, Fig. 1). For the clinical cohort, the image analysis required ~30 minutes to segment and partition the aorta for the five systolic phases per subject per observer. In total, the aortas of the 16 subjects were segmented at five different cardiac phases and then partitioned into five anatomical segments resulting in a total of 1700 individual anatomical aortic lumen segments. The aorta segmentation is described in more detail by Van der Palen et al.^10^

Next, the morphometry of the aorta segmentation was fully automatically processed using in‐house developed Python v3.6.4 (Python Software Foundation, Wilmington, DE) software.^16^, ^17^, ^18^, ^19^ For each anatomical segment, the volume, surface area, centerline length, maximal diameter, and the curvature radius (longitudinal bending radius) were computed. The maximal diameter was derived by first constructing a perpendicular plane to the centerline every millimeter. Next, the cross‐sectional lumen areas between the perpendicular planes and the lumen segmentation were used to derive the lumen diameter, assuming a circular lumen area. The curvature radius was derived by first finding the best‐fitting plane to the segment's centerline. Next, the centerline points were projected on the fitting plane and a circle was fitted through them (see Fig. 1). To determine the accuracy of the in‐house‐developed tool, synthesized and 4D flow MRI phantom data were investigated (see Supplementary S1).

#### *STATISTICAL ANALYSIS*

The statistical analysis of the, interexamination, interobserver reproducibility, and intraobserver repeatability was performed using the SPSS v23 software (IBM, Armonk, NY). All continuous parameters were expressed as the mean with standard deviation (mean ± standard deviation). The characteristic differences within the healthy subject cohort were evaluated with paired *t*‐tests. To assess reproducibility, Bland–Altman analysis,^20^ coefficients of variation (COV), Pearson correlation coefficients (*r*), and intraclass correlation coefficients (ICC) were calculated. For the Bland–Altman analysis the mean difference (Diff) and limits of agreement (LoA; ±1.96 standard deviation of Diff) were computed. The COV was classified as: low (≤10%), intermediate (11–20%), high (21–30%), and very high (>30%). The *r* and ICC were classified as: poor (<0.50), moderate (0.50–0.69), good (0.70–0.84), very good (0.85–0.94), and excellent (≥0.95). *P* < 0.05 was considered statistically significant.

## Results

The baseline characteristics of the healthy volunteers and clinical cohort are shown in Table 2. Between the first and second examinations, the healthy volunteer cohort had no significant differences in heart rate (61 ± 8 vs. 62 ± 6 bpm, *P* = 0.65) and trigger delays for the five systolic phases (*P* = 0.91, 0.86, 0.85, 0.83, and 0.83, respectively, for phases one to five). The phantom analysis demonstrated that the aorta morphometry can be derived from a lumen segmentation with a very‐low (<5%) relative error by the in‐house‐developed software tool (see Supplementary S1). The morphometric baseline characteristics derived from the first 4D flow MRI of all subjects are displayed in Table 3.

**TABLE 2:** Study Population and 4D Flow MRI Characteristics

	Healthy cohort				Clinical cohort		
	Volunteers	First 4D flow MRI	Second 4D flow MRI	Probability value paired *t*‐test	Coarctation patients	Aneurysm patients	Healthy volunteers
Population size	10				2	2	2
Male (%)	5 (50%)				1 (50%)	1 (50%)	1 (50%)
Age [years]	26.5 ± 2.6				13.0 ± 1.4	64.5 ± 7.8	58.5 ± 4.9
Height [cm]	176 ± 7				151 ± 14	181 ± 9	180 ± 1
Weight [kg]	68 ± 3				38 ± 8	80 ± 1	73 ± 16
BSA [m^2^]	1.8 ± 0.2				1.3 ± 0.2	2.0 ± 0.1	1.9 ± 0.2
Heart rate [bpm]		61 ± 8	62 ± 6	0.65	77 ± 18	62 ± 15	64 ± 10
Trigger delay peak systole −2 phases [msec]		104 ± 19	103 ± 19	0.91	122 ± 23	110 ± 38	91 ± 47
Trigger delay peak systole −1 phases [msec]		137 ± 18.4	135 ± 18	0.86	149 ± 25	142 ± 43	120 ± 47
Trigger delay peak systole [msec]		169 ± 18	168 ± 18	0.85	175 ± 25	172 ± 48	149 ± 51
Trigger delay peak systole +1 phases [msec]		202 ± 18	200 ± 17	0.83	202 ± 27	203 ± 52	179 ± 52
Trigger delay peak systole +2 phases [msec]		234 ± 18	232 ± 17	0.83	229 ± 28	235 ± 57	209 ± 54

Data notated as the mean ± standard deviation.

BSA = body surface area according to the DuBois formula; bpm = beats per minute.

**TABLE 3:** Morphometric Characteristics of Study Population

Seg	Cohort	Subgroup	Volume [mL]	Surface area [cm^2^]	Centerline length [mm]	Maximal diameter [mm]	Curvature radius [mm]
pAAo	Healthy	Volunteer	13.6 ± 3.2	22.6 ± 3.9	28.8 ± 4.0	25.7 ± 2.1	33.1 ± 6.3
	Clinical	CoA	11.7 ± 1.6	21.8 ± 0.9	31.9 ± 2.1	24.5 ± 3.7	35.5 ± 5.5
		TAA	49.1 ± 17.1	51.5 ± 14.7	41.8 ± 9.2	42.1 ± 3.8	44.5 ± 3.3
		Volunteer	29.6 ± 10.0	35.2 ± 15.9	45.9 ± 4.6	31.2 ± 4.5	69.2 ± 3.5
dAAo	Healthy	Volunteer	12.7 ± 3.1	20.5 ± 3.7	25.9 ± 3.7	25.7 ± 2.0	37.6 ± 8.4
	Clinical	CoA	9.5 ± 1.1	19.0 ± 1.3	30.1 ± 0.6	21.3 ± 0.3	47.8 ± 17.7
		TAA	50.7 ± 16.8	53.6 ± 15.8	44.6 ± 11.4	41.8 ± 3.8	46.8 ± 13.9
		Volunteer	36.1 ± 12.4	40.1 ± 16.5	46.5 ± 4.6	31.7 ± 4.2	36.1 ± 3.0
AoA	Healthy	Volunteer	12.8 ± 4.1	22.8 ± 5.5	31.3 ± 5.5	24.3 ± 2.0	30.0 ± 8.1
	Clinical	CoA	4.2 ± 1.2	9.9 ± 1.6	18.1 ± 1.1	18.5 ± 2.1	13.3 ± 2.6
		TAA	29.4 ± 1.6	40.3 ± 0.9	43.4 ± 0.3	34.0 ± 0.2	80.6 ± 29.4
		Volunteer	21.0 ± 6.6	27.2 ± 13.1	37.8 ± 7.4	28.9 ± 3.1	59.5 ± 3.1
pDAo	Healthy	Volunteer	10.6 ± 2.6	21.5 ± 4.1	34.3 ± 5.9	21.4 ± 1.8	30.3 ± 5.5
	Clinical	CoA	5.0 ± 0.2	14.2 ± 1.1	31.4 ± 3.8	15.8 ± 1.2	56.4 ± 19.6
		TAA	35.5 ± 0.3	52.0 ± 2.8	58.6 ± 7.8	30.5 ± 3.2	33.3 ± 13.0
		Volunteer	29.7 ± 3.4	39.0 ± 13.1	58.9 ± 4.7	26.5 ± 0.1	35.4 ± 1.5
dDAo	Healthy	Volunteer	8.7 ± 2.0	18.8 ± 2.7	32.1 ± 3.8	19.5 ± 2.2	107.7 ± 49.7
	Clinical	CoA	5.3 ± 1.0	14.7 ± 2.4	32.1 ± 4.6	15.0 ± 0.3	93.5 ± 8.4
		TAA	24.1 ± 3.3	42.2 ± 5.6	58.4 ± 7.7	24.8 ± 0.5	102.4 ± 36.5
		Volunteer	25.8 ± 4.5	35.0 ± 14.2	57.1 ± 4.9	24.7 ± 1.2	196.8 ± 32.9

Characteristics are presented per cohort and subgroup over all cardiac phases and expressed as the mean ± standard deviation.

CoA = coarctation patient, TAA = thoracic ascending aneurysm patient, pAAo = proximal ascending aorta, dAAo = distal ascending aorta, AoA = aortic arch, pDAo = proximal descending aorta, dDAo = distal descending aorta; Seg = segmentation.

**FIGURE 2:**
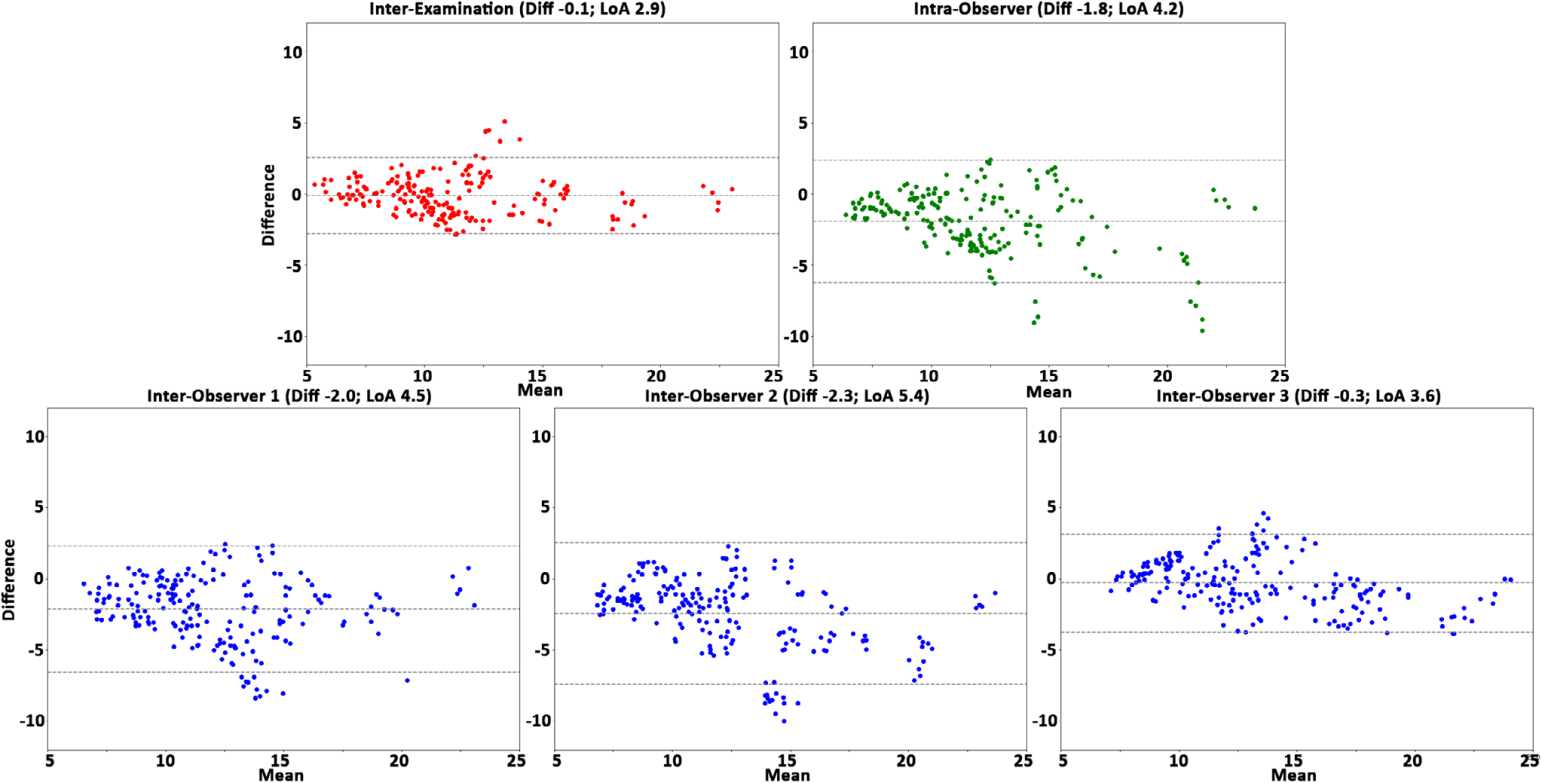
The Bland–Altman plots for volume assessment of the interexamination, intraobserver, and interobserver studies. Plots demonstrate the results over all healthy volunteers, anatomical segments, and cardiac phases (*n =* 250). The plots display volume differences against volume means in mL. Horizontal lines show mean difference (Diff, solid line) and the limits of agreement (LoA, dashed lines).

**FIGURE 3:**
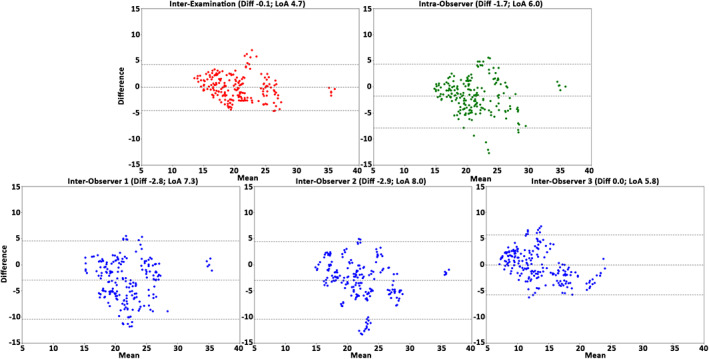
The Bland–Altman plots for surface area assessment of the interexamination, intraobserver, and interobserver studies. Plots demonstrate the results over all healthy volunteers, anatomical segments, and cardiac phases (*n =* 250). Displayed on the vertical and horizontal axis, the difference and means, respectively, in mm^2^. Horizontal lines show the mean difference (Diff, solid line) and the limits of agreement (LoA, dashed lines).

**FIGURE 4:**
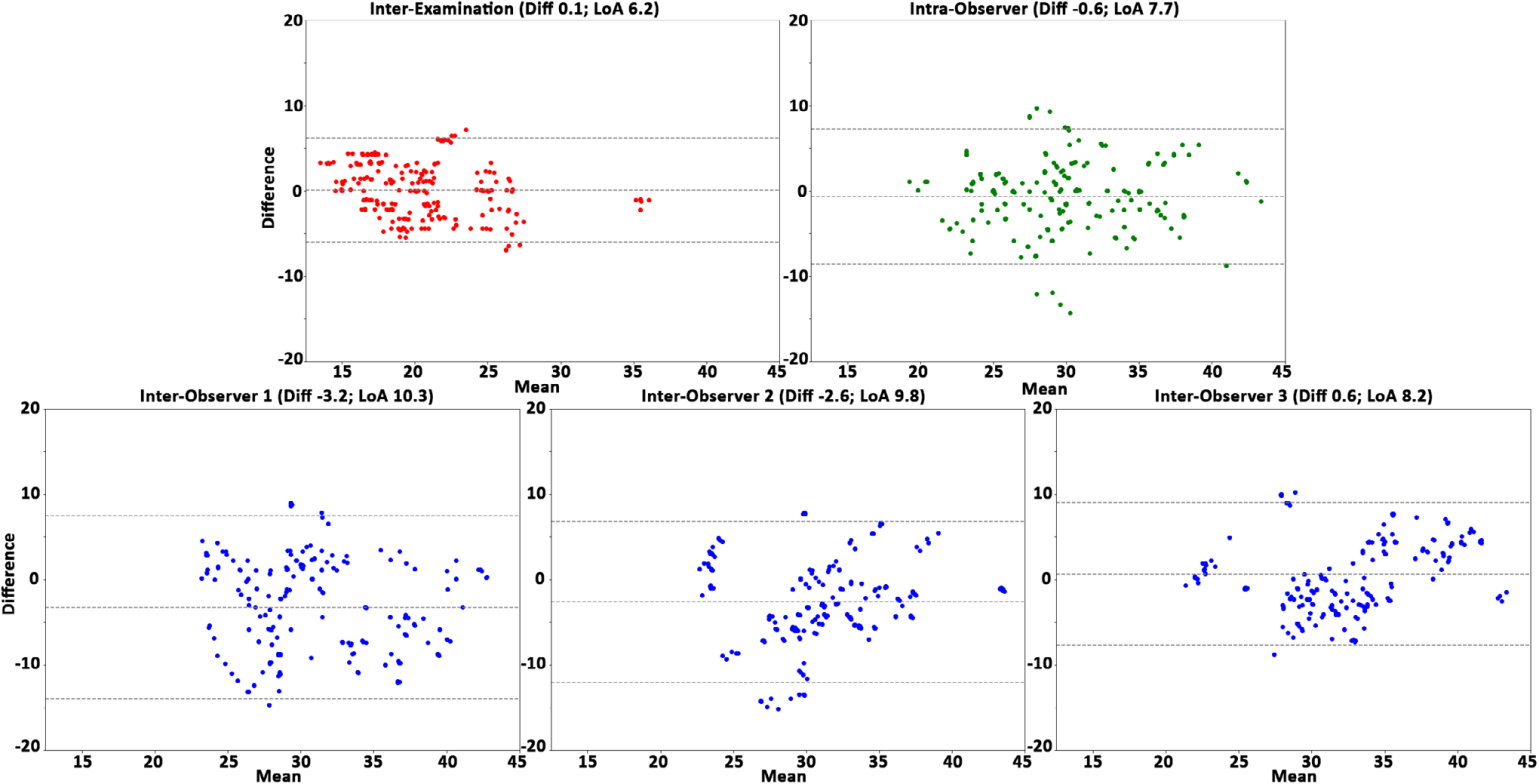
The Bland–Altman plots for centerline length assessment over the interexamination, intraobserver, and interobserver studies. Plots demonstrate the results of all healthy volunteers, anatomical segments, and cardiac phases (*n =* 250). Displayed on the vertical and horizontal axis, the difference and means, respectively, in mm. Horizontal lines show the mean difference (Diff, solid line) and the limits of agreement (LoA, dashed lines).

**FIGURE 5:**
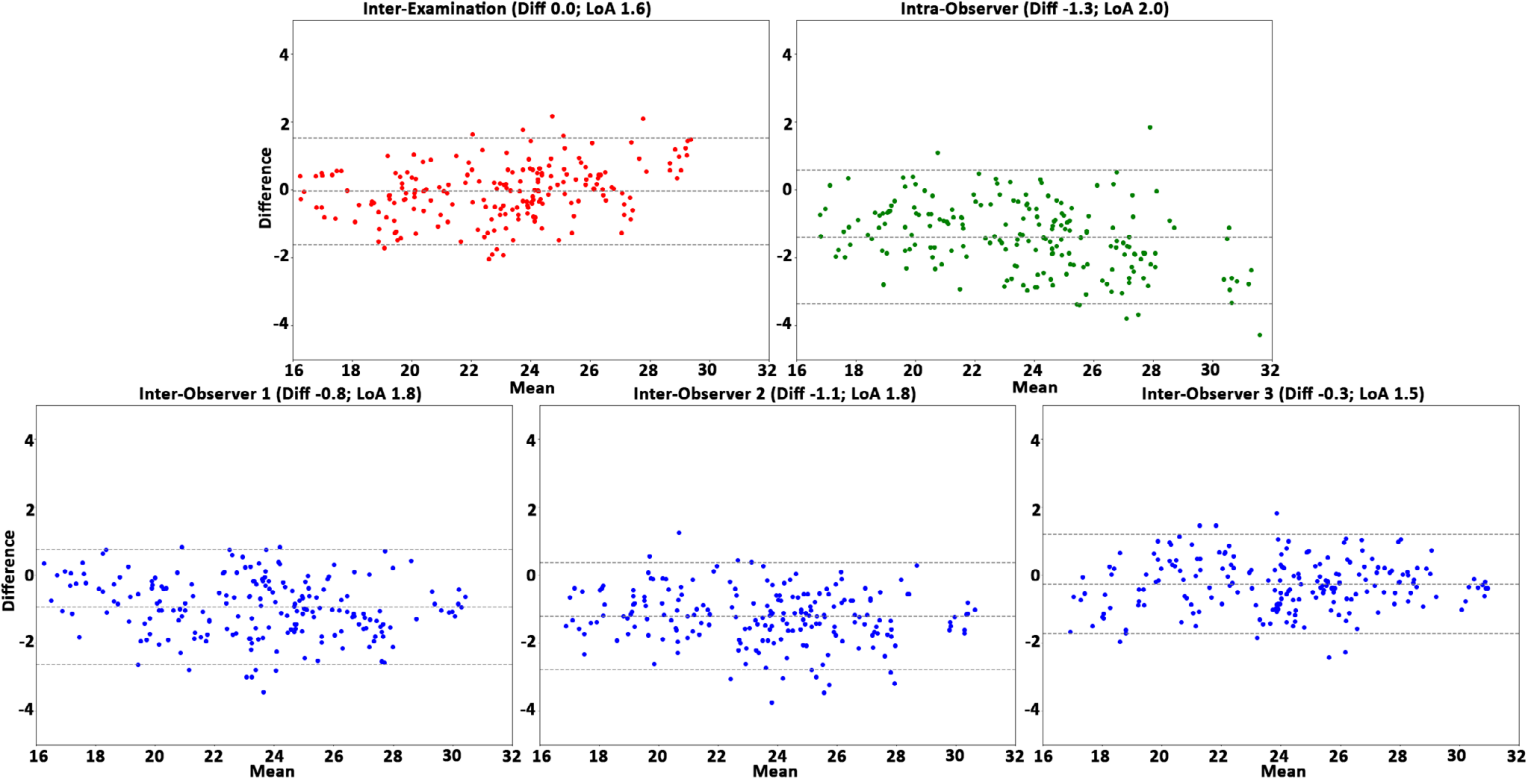
The Bland–Altman plots for the maximal diameter assessment the interexamination, intraobserver, and interobserver studies. Plots demonstrate the results over all healthy volunteers, anatomical segments, and cardiac phases (*n =* 250). Displayed on the vertical and horizontal axis, the difference and means, respectively, in mm. Horizontal lines show the mean difference (Diff, solid line) and the limits of agreement (LoA, dashed lines).

**FIGURE 6:**
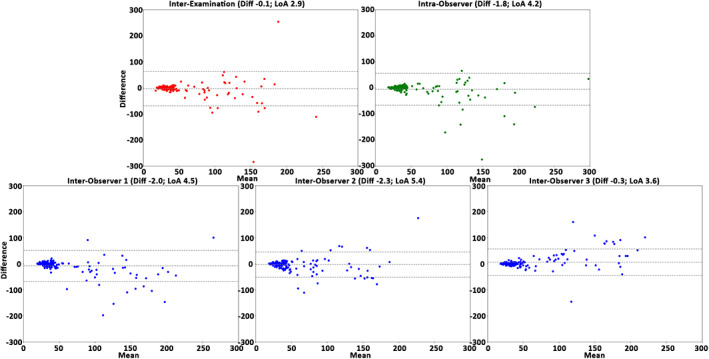
The Bland–Altman plots for the curvature radius assessment of the interexamination, intraobserver, and interobserver studies. Plots demonstrate the results over all healthy volunteers, anatomical segments, and cardiac phases (*n =* 250). Displayed, on the vertical and horizontal axis, the difference and means, respectively, in mm. Horizontal lines show the mean difference (Diff, solid line) and the limits of agreement (LoA, dashed lines).

The interexamination, interobserver reproducibility, and intraobserver repeatability results of the healthy cohort over all subjects, anatomical segments, and cardiac phases are presented in Table 4. In general, for the healthy cohort the analysis demonstrated a very good reproducibility and repeatability for volume, surface area, and centerline length (COV = 10–21%, *r =* 0.54–0.92 and ICC = 0.65–0.96), excellent reproducibility and repeatability for maximal diameter (COV = 3–4%, *r =* 0.96–0.97, ICC = 0.94–0.99), and good reproducibility and repeatability for curvature radius (COV = 44–62%, *r =* 0.73–0.89, ICC = 0.84–0.92). The Bland–Altman plots (Figs. 2, 3, 4, 5, 6) demonstrated LoAs equal to or smaller than: volume 4.5 mL, surface area 7.3 mm^2^, centerline length 10.3 mm, maximal diameter 2.0 mm, and curvature radius 68.0 mm.

**TABLE 4:** Interexamination, Interobserver Reproducibility and Intraobserver Repeatability Results of the Healthy Cohort

	Bland–Altman			Pearson correlation		
Study	Mean Diff	LoA	COV [%]	*r*	*P*	ICC
Volume [cm^3^ = mL]						
IE–E	−0.1	2.9	13	0.92	<0.01	0.96
IA–O	−1.8	4.2	17	0.85	<0.01	0.86
IE–O1	−2.0	4.5	18	0.80	<0.01	0.82
IE‐O2	−2.3	5.4	21	0.78	<0.01	0.79
IE‐O3	−0.3	3.6	13	0.92	<0.01	0.95
Surface area [cm^2^]						
IE–E	−0.1	4.7	11	0.86	<0.01	0.92
IA–O	−1.7	6.0	14	0.75	<0.01	0.82
IE–O1	−2.8	7.3	16	0.61	<0.01	0.66
IE‐O2	−2.9	8.0	18	0.62	<0.01	0.68
IE‐O3	0.0	5.8	12	0.80	<0.01	0.88
Centerline length [mm]						
IE–E	0.1	6.2	10	0.84	<0.01	0.91
IA–O	−0.6	7.7	13	0.73	<0.01	0.84
IE–O1	−3.2	10.3	16	0.60	<0.01	0.68
IE‐O2	−2.6	9.8	16	0.54	<0.01	0.65
IE‐O3	0.6	8.2	13	0.74	<0.01	0.83
Maximal diameter [mm]						
IE–E	0.0	1.6	3	0.97	<0.01	0.98
IA–O	−1.3	2.0	4	0.96	<0.01	0.94
IE–O1	−0.8	1.8	4	0.96	<0.01	0.97
IE‐O2	−1.1	1.8	4	0.96	<0.01	0.95
IE‐O3	−0.3	1.5	3	0.97	<0.01	0.99
Curvature radius [mm]						
IE–E	−3.4	59.9	62	0.73	<0.01	0.84
IA–O	−4.7	56.0	57	0.80	<0.01	0.87
IE–O1	−7.6	54.9	55	0.82	<0.01	0.88
IE‐O2	−3.4	44.5	46	0.82	<0.01	0.90
IE‐O3	4.2	46.0	44	0.89	<0.01	0.92

Results are presented over all healthy volunteers, cardiac phases, and anatomical segments (*n =* 250).

Mean Diff = mean difference, LoA = limits of agreement (1.96 * standard deviation mean difference), COV = coefficient of covariance, *r =* Pearson correlation coefficient, *P* = probability value, ICC = intraclass correlation coefficient, IE‐E = interexamination, IE‐O = interobserver, IA‐O = intraobserver.

The interexamination, interobserver reproducibility, and intraobserver repeatability results of the healthy cohort per anatomical segment are presented in Supplements S2, S3, and S4, respectively. These results showed a comparable reproducibility and repeatability per anatomical segment for most parameters. However, the volume, surface area, and centerline length reproducibility and repeatability were decreased for the proximal ascending aorta (pAAo). The curvature radius reproducibility was decreased for the distal descending aorta (dDAo) (LoA = 90.6–130.9 mm) compared with the other anatomical segments (LoA = 4.7–23.5 mm).

The interobserver reproducibility results of the clinical cohort over all subjects, anatomical segments, and cardiac phases are presented in Table 5. In general, for the clinical cohort the analysis demonstrated a very good reproducibility for volume, surface area, centerline length, and curvature radius (COV = 10–41%, *r =* 0.83–0.95, ICC = 0.91–0.99) and excellent reproducibility for maximal diameter (COV = 4–11%, *r =* 0.94–0.99, ICC = 0.97–0.99). The Bland–Altman analysis demonstrated LoAs equal to or smaller than: volume 14.9 mL, surface area 18.9 mm^2^, centerline length 13.1 mm, maximal diameter 5.8 mm, and curvature radius 54.9 mm.

**TABLE 5:** Interobserver Reproducibility Results of the Clinical Cohort

	Bland–Altman			Pearson correlation		
Study	Mean Diff	LoA	COV [%]	r	*P*	ICC
Volume [cm^3^ = mL]						
IE–O1	−1.0	5.9	12	0.98	<0.01	0.99
IE‐O2	1.2	14.9	32	0.89	<0.01	0.94
IE‐O3	2.2	14.2	30	0.89	<0.01	0.94
Surface area [cm^2^]						
IE–O1	−3.3	11.1	16	0.95	<0.01	0.96
IE‐O2	−0.6	18.9	29	0.83	<0.01	0.90
IE‐O3	2.7	14.2	21	0.89	<0.01	0.93
Centerline length [mm]						
IE–O1	−0.6	8.8	10	0.94	<0.01	0.97
IE‐O2	2.0	13.1	16	0.87	<0.01	0.91
IE‐O3	2.6	11.9	15	0.88	<0.01	0.92
Maximal diameter [mm]						
IE–O1	−0.2	2.5	4	0.98	<0.01	0.99
IE‐O2	0.1	5.1	8	0.93	<0.01	0.96
IE‐O3	0.3	5.4	8	0.93	<0.01	0.96
Curvature radius [mm]						
IE–O1	−0.9	31.1	25	0.95	<0.01	0.97
IE‐O2	4.3	48.1	40	0.85	<0.01	0.90
IE‐O3	5.2	53.6	44	0.84	<0.01	0.89

Results are presented over all subjects, cardiac phases, and anatomical segments (*n =* 150). Mean Diff = mean difference, LoA = limits of agreement – ±1.96. *Standard deviation mean difference.

COV = coefficient of covariance, *r =* correlation coefficient, *P* = probability value, ICC = intraclass correlation coefficient.

The interobserver results of the clinical cohort per subgroup are presented in Supplementary S5. These results showed a comparable reproducibility per subgroup.

## Discussion

In this study the interexamination, interobserver reproducibility, and intraobserver repeatability of aortic lumen segmentation on 4D flow MRI was evaluated in a healthy subject and clinically relevant cohort. The main findings of this study were as follows: 1) The interexamination, interobserver, and intraobserver analysis demonstrated a very good aortic lumen segmentation reproducibility and repeatability. 2) The analysis demonstrated an excellent reproducibility and repeatability for assessment of the maximal diameter. 3) The analysis demonstrated slightly larger, but still acceptable, LoAs for the clinical cohort compared to the healthy cohort.

The interexamination, interobserver, and intraobserver analysis demonstrated comparable reproducibility and repeatability for each parameter for both cohorts. Comparable interobserver reproducibility and intraobserver repeatability have previously been described for volume, centerline length, maximal diameter, and curvature radius in studies analyzing nonelectrocardiographic‐gated contrast‐enhanced MRI, 4D flow MRI, and computed tomography images in patients.^21^, ^22^, ^23^, ^24^, ^25^

The reproducibility results of the current study demonstrated no major limitations for the (semi‐)automatic aortic lumen segmentation of 4D flow MRI. However, some segmentation variability was observed that will affect the reproducibility and repeatability of flow‐derived parameters (eg, quantification of kinetic energy for a specific volume^26^). The Bland–Altman plots demonstrated consistent differences over a range of values for all morphometric parameters except for the curvature radius. In order to obtain the smallest relative error, it is recommended that aortic morphometrics and flow‐derived parameters are derived over a large anatomical segment.

However, for the clinical cohort slightly larger, but still acceptable, LoAs were observed compared to the healthy cohort. For patients and older subjects, more vessel irregularity, complex flow patterns, heart rate variance, and breathing variance is expected compared to healthy volunteers and young subjects. These clinical cohort characteristics potentially reduce the velocity‐to‐noise of the 4D flow MRI, which possibly decreased the reproducibility. But, with the use of a multiple‐velocity‐encoding and highly accelerated sequences, the velocity‐to‐noise of the 4D flow MRI can potentially be improved and possibly improves the segmentation reproducibility.^27^, ^28^, ^29^

Furthermore, for the healthy and clinical cohort the Bland–Altman analysis demonstrated that the interexamination, interobserver, and intraobserver LoAs for the maximal diameter were 1.5–2.0 mm and 2.4–5.8 mm, respectively. These LoAs for the clinical cohort were below the spatial resolution of the 4D flow MRI. Due to variation in manually repeated measurements of maximal aortic diameters, the European Society of Cardiology considers a change in maximal aortic diameter larger than 5 mm as significant.^14^ Hence, the image analysis method for deriving maximal lumen diameter used in this study could have the potential to describe patient characteristics and would be beneficial for the clinical follow‐up of patients with pathological aorta diameters, like aneurysms or coarctations.

The low LoAs for the maximal diameter, especially in the healthy cohorts, is presumably driven by the automatic diameter analysis utilized in this study, which determines the maximal diameter over an anatomical segment by constructing a perpendicular plane every millimeter along the segmentation's centerline. The automatic analysis is less influenced by the observer than the manual measurement method, where the observer potentially chooses different measurement locations and plane obliquity towards the vessel.^15^ Eventually, both intra‐ and interobserver variability may potentially be removed by the application of a fully automated 4D flow MRI segmentation method, although interexamination variability would remain.^25^, ^30^ However, these applications are currently only able to create a single segmentation from a time‐averaged 4D flow MRI. For moving and stretching vessels (eg, ascending aorta), these time‐averaged segmentations can have a misalignment between the segmentation and actual lumen border for specific phases. This misalignment could potentially be problematic when calculating time‐specific patient flow properties.

When analyzing the healthy cohort results per anatomical segment, it was observed that the volume, surface area, and centerline length reproducibility was decreased for the pAAo. This may be explained by the minimal anatomic landmark information recorded within the 4D flow MRI, resulting in difficulties in positioning the most proximal plane at the sinotubular junction.^10^ The reduced pAAo reproducibility may also be explained by the pronounced longitudinal stretching and movement of the ascending aorta during systole compared to the aortic arch and descending aorta.^23^, ^31^, ^32^ A decreased reproducibility was also observed for the dDAo curvature radius. This may be explained by the minimal longitudinal bending of the dDAo, resulting in instabilities when trying to fit a circle to a nearly straight centerline. This reasoning is supported by the considerably larger curvature radii observed in dDAo and the Bland–Altman plots, which demonstrate higher differences for larger radii.

## Limitations

This study incorporated a healthy volunteer and clinical cohort of only ten and six subjects, respectively. The relatively larger group of healthy volunteers in the study population has probably a less varying heart rate and breathing pattern, which presumably contributed to a better image quality compared to clinical patients. The cohorts, including the subgroups, also had a relatively small age range. Also, only two types of patients with pathological aortas were evaluated, excluding the possibility of verifying the segmentation reproducibility in other pathological aortas.

However, the thoracic aorta lumen was segmented by three observers for five cardiac phases and then partitioned into five anatomical segments, which resulted in a total of 1700 aortic lumen segments, which improved the robustness of the study.

## Conclusion

This study demonstrated no major reproducibility and repeatability limitations for 4D flow MRI aortic lumen segmentation.

## Supporting information


**Supplementary Figure S1** The phantom lumen partitioning.
**Supplementary S1 Table 1** The phantom accuracy results over all five segments, presented as percentage of the mismatch divided by the true value.
**Supplementary S1 Table 2** The phantom accuracy results per segment.
**Supplementary S2 Table.** The interexamination reproducibility results of the healthy cohort per anatomical segment.
**Supplementary S3 Table.** The interobserver reproducibility results of the healthy cohort per anatomical segment.
**Supplementary S4 Table.** The intraobserver reproducibility results of the healthy cohort per anatomical segment.
**Supplementary S5** The interobserver results of the clinical cohort per subgroup.
