# Supplementary material for: Reproducibility of Aorta Segmentation on 4D Flow MRI in Healthy Volunteers
Source: J Magn Reson Imaging. 2020 Nov 11;53(4):1268–79. doi: 10.1002/jmri.27431 (PMC7984392; doi:10.1002/jmri.27431)
Supplement: Supplementary file 1 — Supplementary Figure S1 The phantom lumen partitioning. Supplementary S1 Table 1 The phantom accuracy results over all five segments, presented as percentage of the mismatch divided by the true value. Supplementary S1 Table 2 The phantom accuracy results per segment. Supplementary S2 Table. The interexamination reproducibility results of the healthy cohort per anatomical segment. Supplementary S3 Table. The interobserver reproducibility results of the healthy cohort per anatomical segment. Supplementary S4 Table. The intraobserver reproducibility results of the healthy cohort per anatomical segment. Supplementary S5 The interobserver results of the clinical cohort per subgroup. [file JMRI-53-1268-s001.docx]

**SUPPLEMENT 1.** The accuracy analysis of the in-house developed tool and 4D flow analysis.

**INTRODUCTION**

The in-house developed software tool to analyze the aorta morphometry was programmed in Python v3.6.4 (Python Software Foundation, Welfeboro Falls, USA) and utilized several open-source scientific libraries: SciPy v.1.1.1 (16), NumPy v1.12.1 (17), Visualization ToolKit v.8.1. (18) and Vascular Modeling ToolKit v1.4.0 (19). To assess the inaccuracy of this software tool and 4D flow MRI analysis, a simulated phantom segmentation and 4D flow MRI phantom data were incorporated as a reference respectively. From a U-shaped phantom blueprint including a distal narrowing (Supplement 1 Figure 1), a simulated phantom segmentation and an MRI-compatible flow phantom were created.

**MATERIALS AND METHODS**

The simulated phantom segmentation was directly analyzed with the in-house developed software. By manually placing anatomical planes the phantom lumen was divided into five consecutive segments: the inlet tube, U-bend, proximal outlet tube, stenosis tube and the distal outlet tube (Supplement 1 Figure 1).

The MRI-compatible flow phantom (Materialise, Leuven, Belgium) was submerged in a tank filled with a combined gadobutrol (Gadovist®, Bayer Pharma, Berlin, Germany) and gelatin composition to increase signal intensity. The exact composition consisted of 9.9 L water, 600 g gelatin, 100 mL paraben and 1.5 mL gadobutrol. The composition was circulated through the phantom using a stationary pump (AQUA F 10 L, Fiamma, Varese, Italy) with two different settings to assess the effect of different velocities. The U-shaped phantom was scanned in a sagittal imaging direction, resulting in parallel planes towards the legs of the phantom respectively (Supplement 1 Figure 1). The acquisition settings were; (1) flow rate 4.5 L/min, VENC 70 cm/s. (2) flow rate 5.7 L/min, VENC 120 cm/s. None of the acquired 4D flow MRI data sets was subject to phase-wrapping.

The 4D flow MRI was acquired using retrospective electrocardiographic gating with a simulated heart rate of 60 bpm. Sequence parameters were as follows: acquisition spatial resolution: 1.5 × 1.5 × 1.5 mm^3^, reconstruction spatial resolution: 0.7 x 0.7 x 1.5 mm^3^, temporal resolution: 33-50 ms (28 – 30 phases), echo time/repetition time: 2.3–2.5 ms/4.0–4.3 ms, flip angle: 7°, field of view: 180 × 180 × 40.5 mm^3^, turbo field echo factor: 2, sensitivity encoding factor 2 in anterior–posterior direction. Concomitant gradient correction and local phase correction were performed from standard available scanner software. Next, the phantom 4D flow MRI data was semi-automatically segmented by a single observer (JJ) utilizing CAAS MR Solutions v5.0 (Pie Medical Imaging, Maastricht, The Netherlands). By manually placing start and end points in the phantom, automatically a lumen segmentation was created which subsequently manually was improved.

The phantom segmentation was analyzed with the in-house developed software for the same five segments used for the synthesized phantom segmentation. From the simulated and 4D flow MRI phantom segmentation, the morphometric parameters were computed for each anatomical segment by the in-house developed software. To assess the inaccuracy of the in-house developed software and 4D flow MRI analysis the relative error was determined (calculated as the mismatch between the observed value and true value divided by the true valve, and expressed as percentage). For volume and surface area, this calculation was implemented using the computed centerline length and the true diameter of each segment. The relative error was classified as: very low (≤5%), low (6-10%), intermediate (11–20%), high (21–30%) and very high (>30%).

**RESULTS**

The simulated phantom segmentation inaccuracy analysis with the in-house developed software tool showed a very low relative error for all parameters, see Supplement 1 Table 1. Between the two segmentations comparable inaccuracies were found over all five segments, the analysis revealed very low relative errors for all parameters, except for volume, which showed a low relative error (volume: 5.7 – 6.2%, surface area: 2.9 – 3.2%, mean diameter: 2.8 – 2.9%, maximal diameter: 3.6 – 4.1% and curvature: -1.2 – 0.0%). Furthermore, the 4D flow MRI segmentation analysis demonstrated for most parameters an overestimation of the blueprint dimensions, see Supplement 1 Table 1.

When analyzing the separate anatomical segments, comparable results were found for most morphometric parameters, see Supplement 1 Table 2. Only in the U-bend segment an increased inaccuracy was found for the volume and surface area, demonstrating an intermediate-to-low relative errors (volume 10.5 – 13.5% and surface area 7.0 – 10.0%).

**DISCUSSION**

The phantom analysis demonstrated that the in-house developed software tool derived the morphometric parameters from the simulated phantom segmentation with a very low relative error, even while the phantom’s lumen was manually partitioned in multiple anatomical segments. Additionally, the MRI-compatible flow phantom analysis also demonstrated that the 4D flow MRI segmentation matched the phantom’s blueprint mostly with a very low relatively. This demonstrated a low inaccuracy for semi-automatic lumen segmentation. However, the segmentation and partitioning of the phantom lumen is easier to perform compared to a human aorta, due to the regular shape and clear geometry transitions of the phantom. Therefore, larger observer errors were expected during the image analysis of a human 4D flow MRI compared to the phantom 4D flow MRI.

**CONCLUSION**

The phantom analysis demonstrated a very-low inaccuracy for both the in-house developed software tool and the 4D flow MRI analysis for the morphometry quantification.

**Supplement 1 Figure 1.** The phantom lumen partitioning.


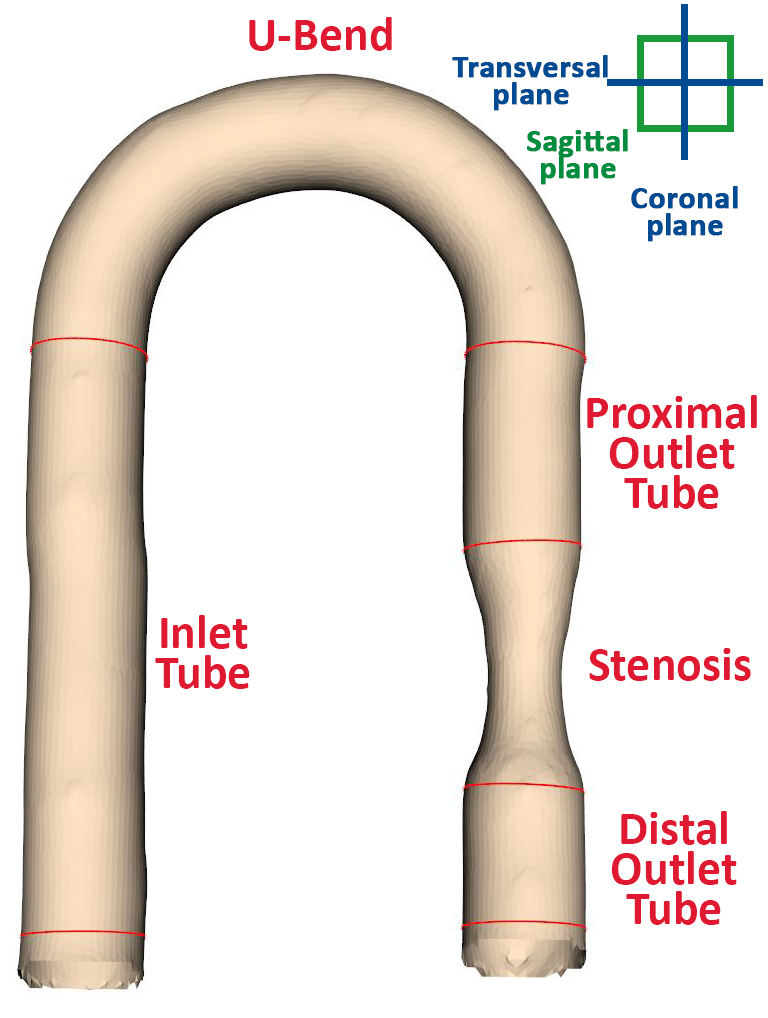


**SUPPLEMENT 1 TABLE 1.** The phantom accuracy results over all five segments, presented as percentage of the mismatch divided by the true value.

| Seg | Pump Setting | Volume [%] | Surface Area [%] | Mean Diameter [%] | Max Diameter [%] | Curvature Radius [%] |
| --- | --- | --- | --- | --- | --- | --- |
| Syn | x | 0.0 [0.0 – 0.0] | 0.0 [0.0 – 0.0] | 0.0 [0.0 – 0.0] | 0.0 [0.0 – 0.0] | 0.0 |
| 4D flow MRI | Low | 5.7 [4.1 – 6.8] | 2.9 [2.2 – 3.5] | 2.9 [2.2 – 3.3] | 4.1 [3.1 – 4.1] | -1.2 |
|  | High | 6.2 [5.5 – 6.8] | 3.2 [2.8 – 3.4] | 2.8 [0.1 – 3.1] | 3.6 [3.4 – 4.3] | 0.0 |

Data presented as percentage of the mismatch between the observed value and true value divided by the true valve, and expressed as percentage, over all segments (n=5) and notated as the median, lower and upper quartile (median [lower quartile – upper quartile). Abbreviations: Syn – synthetically constructed phantom, and x – not applicable.

**SUPPLEMENT 1 TABLE 2.** The phantom accuracy results per segment.

| Inlet Tube | | | | | |
| --- | --- | --- | --- | --- | --- |
| Pump Setting | Volume [%] | Surface Area [%] | Mean Diameter [%] | Max Diameter [%] |  |
| Low | 5.7 | 2.9 | 2.8 | 5.6 |  |
| High | 6.8 | 3.4 | 0.1 | 4.5 |  |
| U-Bend | | | | | |
| Pump Setting | Volume [%] | Surface Area [%] | Mean Diameter [%] | Max Diameter [%] | Curvature Radius [%] |
| Low | 13.5 | 10.0 | 3.3 | 4.1 | -1.2 |
| High | 10.5 | 7.0 | 3.3 | 4.3 | 0.0 |
| Proximal Outlet Tube | | | | | |
| Pump Setting | Volume [%] | Surface Area [%] | Mean Diameter [%] | Max Diameter [%] |  |
| Low | 4.1 | 2.2 | 2.2 | 3.1 |  |
| High | 5.5 | 2.8 | 2.8 | 3.5 |  |
| Stenosis | | | | | |
| Pump Setting | Volume [%] | Surface Area [%] | Mean Diameter [%] | Max Diameter [%] |  |
| Low | 0.2 | 0.8 | -1.6 | -0.4 |  |
| High | -3.6 | -0.8 | -3.4 | -0.4 |  |
| Distal Outlet Tube | | | | | |
| Pump Setting | Volume [%] | Surface Area [%] | Mean Diameter [%] | Max Diameter [%] |  |
| Low | 6.8 | 3.5 | 3.3 | 4.1 |  |
| High | 6.2 | 3.2 | 3.1 | 3.4 |  |

Data presented as percentage of the mismatch between the observed value and true value divided by the true valve, and expressed as percentage

**SUPPLEMENT 2 – TABLE.** The inter-examination reproducibility results of the healthy cohort per anatomical segment.

| Volume [cm^3^ = mL] | | | | | | |
| --- | --- | --- | --- | --- | --- | --- |
| Seg | Bland–Altman | | COV [%] | Pearson correlation | | ICC |
|  | Mean Diff | LoA |  | r | p |  |
| pAAo | 0.4 | 3.9 | 15 | 0.81 | <0.01 | 0.90 |
| dAAo | -0.7 | 3.4 | 13 | 0.89 | <0.01 | 0.92 |
| AoA | 0.3 | 2.4 | 10 | 0.96 | <0.01 | 0.98 |
| pDAo | -0.2 | 2.3 | 11 | 0.90 | <0.01 | 0.95 |
| dDAo | -0.4 | 1.6 | 9 | 0.94 | <0.01 | 0.96 |
| Surface Area [cm^2^] | | | | | | |
| Seg | Bland–Altman | | COV [%] | Pearson correlation | | ICC |
|  | Mean Diff | LoA |  | r | p |  |
| pAAo | 0.9 | 6.2 | 14 | 0.66 | <0.01 | 0.79 |
| dAAo | -1.0 | 4.9 | 12 | 0.84 | <0.01 | 0.89 |
| AoA | 0.4 | 3.7 | 8 | 0.95 | <0.01 | 0.97 |
| pDAo | -0.2 | 3.9 | 9 | 0.89 | <0.01 | 0.94 |
| dDAo | -0.5 | 3.0 | 8 | 0.88 | <0.01 | 0.92 |
| Centerline Length [mm] | | | | | | |
| Seg | Bland–Altman | | COV [%] | Pearson correlation | | ICC |
|  | Mean Diff | LoA |  | r | p |  |
| pAAo | 1.4 | 7.8 | 14 | 0.42 | <0.01 | 0.56 |
| dAAo | -1.3 | 5.7 | 11 | 0.75 | <0.01 | 0.83 |
| AoA | 0.7 | 5.0 | 8 | 0.91 | <0.01 | 0.95 |
| pDAo | 0.0 | 5.4 | 8 | 0.90 | <0.01 | 0.95 |
| dDAo | -0.5 | 5.3 | 8 | 0.77 | <0.01 | 0.87 |
| Maximal Diameter [mm] | | | | | | |
| Seg | Bland-Altman | |  | Pearson correlation | | ICC |
|  | Mean Diff | LoA | COV [%] | r | p |  |
| pAAo | 0.0 | 1.6 | 3 | 0.92 | <0.01 | 0.96 |
| dAAo | 0.0 | 1.5 | 3 | 0.93 | <0.01 | 0.96 |
| AoA | 0.2 | 1.6 | 3 | 0.91 | <0.01 | 0.95 |
| pDAo | -0.1 | 1.8 | 4 | 0.86 | <0.01 | 0.93 |
| dDAo | -0.1 | 1.4 | 4 | 0.95 | <0.01 | 0.97 |
| Curvature Radius [mm] | | | | | | |
| Study | Bland–Altman | | COV [%] | Pearson correlation | | ICC |
|  | Mean Diff | LoA |  | r | p |  |
| pAAo | 0.7 | 9.2 | 14 | 0.72 | <0.01 | 0.83 |
| dAAo | -1.3 | 10.8 | 15 | 0.79 | <0.01 | 0.88 |
| AoA | 0.9 | 7.8 | 14 | 0.86 | <0.01 | 0.92 |
| pDAo | -2.7 | 13.7 | 22 | 0.76 | <0.01 | 0.75 |
| dDAo | -14.4 | 130.9 | 58 | 0.20 | 0.161 | 0.33 |

Data presented over all healthy volunteers and cardiac phases (n=50). Abbreviations: Mean Diff –mean difference, Limits of agreement – ±1.96 * standard deviation mean difference, COV – coefficient of covariance, r –correlation coefficient, p – probability value, ICC – intra-class correlation coefficient, pAAo – proximal ascending aorta, dAAo – distal ascending aorta, AoA – Aortic arch, pDAo – proximal descending aorta and dDAo – distal descending aorta.

**SUPPLEMENT 3 – TABLE.** The inter-observer reproducibility results of the healthy cohort per anatomical segment.

| Volume [cm^3^ = mL] | | | | | | | |
| --- | --- | --- | --- | --- | --- | --- | --- |
| Seg | Analysis | Bland–Altman | | COV [%] | Pearson correlation | | ICC |
|  |  | Mean Diff | LoA |  | r | p |  |
| pAAo | 1 | -1.4 | 5.1 | 18 | 0.69 | <0.01 | 0.78 |
|  | 2 | -3.1 | 6.1 | 21 | 0.59 | <0.01 | 0.59 |
|  | 3 | -1.7 | 2.3 | 7 | 0.95 | <0.01 | 0.91 |
| dAAo | 1 | -3.4 | 5.2 | 18 | 0.63 | <0.01 | 0.57 |
|  | 2 | -5.3 | 4.9 | 16 | 0.71 | <0.01 | 0.46 |
|  | 3 | -1.9 | 2.2 | 6 | 0.95 | <0.01 | 0.89 |
| AoA | 1 | -0.2 | 2.8 | 11 | 0.94 | <0.01 | 0.97 |
|  | 2 | -0.2 | 3.1 | 12 | 0.95 | <0.01 | 0.97 |
|  | 3 | 0.0 | 2.4 | 9 | 0.97 | <0.01 | 0.98 |
| pDAo | 1 | -2.5 | 3.5 | 15 | 0.79 | <0.01 | 0.71 |
|  | 2 | -0.9 | 3.4 | 16 | 0.76 | <0.01 | 0.82 |
|  | 3 | 1.6 | 2.8 | 12 | 0.87 | <0.01 | 0.83 |
| dDAo | 1 | -2.5 | 2.2 | 13 | 0.89 | <0.01 | 0.70 |
|  | 2 | -1.9 | 2.0 | 11 | 0.89 | <0.01 | 0.77 |
|  | 3 | 0.6 | 1.2 | 11 | 0.87 | <0.01 | 0.91 |
| Surface Area [cm^2^] | | | | | | | |
| Seg | Analysis | Bland–Altman | | COV [%] | Pearson correlation | | ICC |
|  |  | Mean Diff | LoA |  | r | p |  |
| pAAo | 1 | -1.6 | 7.2 | 16 | 0.50 | <0.01 | 0.63 |
|  | 2 | -4.1 | 8.0 | 17 | 0.41 | <0.01 | 0.41 |
|  | 3 | -2.5 | 3.3 | 7 | 0.89 | <0.01 | 0.84 |
| dAAo | 1 | -4.5 | 7.9 | 18 | 0.33 | 0.018 | 0.31 |
|  | 2 | -7.2 | 7.1 | 15 | 0.49 | <0.01 | 0.28 |
|  | 3 | -2.7 | 3.1 | 6 | 0.90 | <0.01 | 0.82 |
| AoA | 1 | 0.9 | 4.3 | 10 | 0.92 | <0.01 | 0.95 |
|  | 2 | 0.8 | 4.3 | 10 | 0.94 | <0.01 | 0.96 |
|  | 3 | -0.1 | 3.5 | 8 | 0.96 | <0.01 | 0.98 |
| pDAo | 1 | -4.0 | 5.7 | 12 | 0.73 | <0.01 | 0.64 |
|  | 2 | -1.0 | 5.8 | 13 | 0.70 | <0.01 | 0.79 |
|  | 3 | 3.0 | 4.3 | 9 | 0.80 | <0.01 | 0.73 |
| dDAo | 1 | -4.9 | 3.7 | 9 | 0.80 | <0.01 | 0.49 |
|  | 2 | -2.8 | 3.3 | 8 | 0.87 | <0.01 | 0.75 |
|  | 3 | 2.1 | 3.9 | 6 | 0.81 | <0.01 | 0.80 |
| Centerline Length [mm] | | | | | | | |
| Seg | Analysis | Bland–Altman | | COV [%] | Pearson correlation | | ICC |
|  |  | Mean Diff | LoA |  | r | p |  |
| pAAo | 1 | -1.4 | 8.8 | 15 | 0.15 | 0.30 | 0.24 |
|  | 2 | -4.5 | 8.5 | 14 | 0.22 | 0.13 | 0.20 |
|  | 3 | -3.0 | 4.6 | 7 | 0.66 | <0.01 | 0.59 |
| dAAo | 1 | -4.6 | 9.7 | 18 | -0.17 | 0.23 | -0.19 |
|  | 2 | -7.6 | 8.5 | 15 | 0.11 | 0.43 | 0.06 |
|  | 3 | -3.0 | 3.6 | 6 | 0.78 | <0.01 | 0.66 |
| AoA | 1 | 2.5 | 5.8 | 10 | 0.86 | <0.01 | 0.88 |
|  | 2 | 2.2 | 5.4 | 9 | 0.91 | <0.01 | 0.91 |
|  | 3 | -0.3 | 4.3 | 8 | 0.95 | <0.01 | 0.97 |
| pDAo | 1 | -5.0 | 8.0 | 11 | 0.73 | <0.01 | 0.62 |
|  | 2 | -0.5 | 8.5 | 13 | 0.69 | <0.01 | 0.79 |
|  | 3 | 4.5 | 5.3 | 7 | 0.80 | <0.01 | 0.66 |
| dDAo | 1 | -7.5 | 5.2 | 8 | 0.74 | <0.01 | 0.39 |
|  | 2 | -2.7 | 4.6 | 7 | 0.89 | <0.01 | 0.84 |
|  | 3 | 4.8 | 5.2 | 7 | 0.86 | <0.01 | 0.67 |
| Maximal Diameter [mm] | | | | | | | |
| Seg | Analysis | Bland-Altman | |  | Pearson correlation | | ICC |
|  |  | Mean Diff | LoA | COV [%] | r | p |  |
| pAAo | 1 | -0.8 | 1.9 | 4 | 0.90 | <0.01 | 0.92 |
|  | 2 | -1.3 | 2.1 | 4 | 0.86 | <0.01 | 0.84 |
|  | 3 | -0.5 | 1.6 | 3 | 0.93 | <0.01 | 0.95 |
| dAAo | 1 | -1.0 | 1.7 | 3 | 0.91 | <0.01 | 0.90 |
|  | 2 | -1.3 | 1.7 | 3 | 0.91 | <0.01 | 0.86 |
|  | 3 | -0.3 | 1.0 | 2 | 0.97 | <0.01 | 0.98 |
| AoA | 1 | -0.9 | 1.6 | 3 | 0.91 | <0.01 | 0.90 |
|  | 2 | -1.1 | 1.6 | 3 | 0.91 | <0.01 | 0.87 |
|  | 3 | -0.2 | 1.3 | 3 | 0.93 | <0.01 | 0.96 |
| pDAo | 1 | -0.8 | 2.1 | 5 | 0.84 | <0.01 | 0.86 |
|  | 2 | -0.7 | 1.7 | 4 | 0.90 | <0.01 | 0.92 |
|  | 3 | 0.2 | 1.8 | 4 | 0.89 | <0.01 | 0.94 |
| dDAo | 1 | -0.5 | 1.5 | 4 | 0.94 | <0.01 | 0.95 |
|  | 2 | -1.1 | 1.4 | 4 | 0.94 | <0.01 | 0.92 |
|  | 3 | -0.6 | 1.3 | 3 | 0.95 | <0.01 | 0.96 |
| Curvature Radius [mm] | | | | | | | |
| Study | Analysis | Bland–Altman | | COV [%] | Pearson correlation | | ICC |
|  |  | Mean Diff | LoA |  | r | P |  |
| pAAo | 1 | -3.9 | 17.2 | 25 | 0.27 | 0.06 | 0.37 |
|  | 2 | -7.4 | 23.5 | 34 | 0.27 | 0.06 | 0.28 |
|  | 3 | -3.5 | 18.2 | 24 | 0.64 | <0.01 | 0.73 |
| dAAo | 1 | -0.7 | 11.8 | 15 | 0.79 | <0.01 | 0.88 |
|  | 2 | -0.2 | 11.4 | 16 | 0.70 | <0.01 | 0.79 |
|  | 3 | 0.5 | 11.2 | 15 | 0.77 | <0.01 | 0.82 |
| AoA | 1 | 1.3 | 9.6 | 17 | 0.79 | <0.01 | 0.88 |
|  | 2 | 0.0 | 9.1 | 16 | 0.85 | <0.01 | 0.92 |
|  | 3 | -1.3 | 8.1 | 14 | 0.88 | <0.01 | 0.93 |
| pDAo | 1 | -2.7 | 11.7 | 19 | 0.48 | <0.01 | 0.60 |
|  | 2 | -1.6 | 11.5 | 19 | 0.46 | <0.01 | 0.61 |
|  | 3 | 1.1 | 4.7 | 7 | 0.92 | <0.01 | 0.95 |
| dDAo | 1 | -31.7 | 108.3 | 51 | 0.43 | <0.01 | 0.54 |
|  | 2 | -7.6 | 94.5 | 43 | 0.45 | <0.01 | 0.61 |
|  | 3 | 24.1 | 90.6 | 36 | 0.54 | <0.01 | 0.63 |

Data presented over all healthy volunteers and cardiac phases (n=50). Abbreviations: Mean Diff –mean difference, Limits of agreement – ±1.96 * standard deviation mean difference, COV – coefficient of covariance, r –correlation coefficient, P – probability value, ICC – intra-class correlation coefficient, pAAo – proximal ascending aorta, dAAo – distal ascending aorta, AoA – Aortic arch, pDAo – proximal descending aorta and dDAo – distal descending aorta.

**SUPPLEMENT 4 – TABLE.** The intra-observer reproducibility results of the healthy cohort per anatomical segment.

| Volume [cm^3^ = mL] | | | | | | |
| --- | --- | --- | --- | --- | --- | --- |
| Seg | Bland–Altman | | COV [%] | Pearson correlation | | ICC |
|  | Mean Diff | LoA |  | r | p |  |
| pAAo | -2.2 | 5.5 | 19 | 0.72 | <0.01 | 0.75 |
| dAAo | -2.4 | 4.3 | 16 | 0.84 | <0.01 | 0.80 |
| AoA | -0.6 | 3.2 | 13 | 0.93 | <0.01 | 0.96 |
| pDAo | -2.0 | 4.4 | 19 | 0.70 | <0.01 | 0.72 |
| dDAo | -2.0 | 2.1 | 11 | 0.93 | <0.01 | 0.79 |
| Surface Area [cm^2^] | | | | | | |
| Seg | Bland–Altman | | COV [%] | Pearson correlation | | ICC |
|  | Mean Diff | LoA |  | r | p |  |
| pAAo | -1.0 | 7.1 | 16 | 0.58 | <0.01 | 0.72 |
| dAAo | -2.3 | 2.9 | 13 | 0.76 | <0.01 | 0.79 |
| AoA | 0.0 | 4.8 | 11 | 0.90 | <0.01 | 0.95 |
| pDAo | -2.2 | 7.1 | 16 | 0.60 | <0.01 | 0.68 |
| dDAo | -2.8 | 3.1 | 8 | 0.88 | <0.01 | 0.75 |
| Centerline Length [mm] | | | | | | |
| Seg | Bland–Altman | | COV [%] | Pearson correlation | | ICC |
|  | Mean Diff | LoA |  | r | p |  |
| pAAo | 1.2 | 7.9 | 14 | 0.39 | <0.01 | 0.54 |
| dAAo | -1.4 | 6.3 | 12 | 0.65 | <0.01 | 0.76 |
| AoA | 0.9 | 6.2 | 10 | 0.83 | <0.01 | 0.90 |
| pDAo | -1.3 | 10.0 | 15 | 0.53 | <0.01 | 0.64 |
| dDAo | -2.7 | 4.6 | 7 | 0.79 | <0.01 | 0.76 |
| Maximal Diameter [mm] | | | | | | |
| Seg | Bland-Altman | |  | Pearson correlation | | ICC |
|  | Mean Diff | LoA | COV [%] | r | P |  |
| pAAo | -2.0 | 2.0 | 4 | 0.90 | <0.01 | 0.78 |
| dAAo | -1.8 | 1.7 | 3 | 0.93 | <0.01 | 0.81 |
| AoA | -0.7 | 1.7 | 4 | 0.91 | <0.01 | 0.92 |
| pDAo | -0.9 | 1.8 | 4 | 0.90 | <0.01 | 0.90 |
| dDAo | -1.2 | 1.6 | 4 | 0.94 | <0.01 | 0.90 |
| Curvature Radius [mm] | | | | | | |
| Seg | Bland–Altman | | COV [%] | Pearson correlation | | ICC |
|  | Mean Diff | LoA |  | r | p |  |
| pAAo | -0.1 | 9.4 | 15 | 0.69 | <0.01 | 0.82 |
| dAAo | 0.5 | 12.0 | 17 | 0.69 | <0.01 | 0.82 |
| AoA | 0.3 | 7.7 | 13 | 0.86 | <0.01 | 0.93 |
| pDAo | -1.8 | 12.7 | 21 | 0.50 | <0.01 | 0.63 |
| dDAo | -22.5 | 118.0 | 50 | 0.39 | <0.01 | 0.53 |

Data presented over all healthy volunteers and cardiac phases (n=50). Abbreviations: Mean Diff –mean difference, Limits of agreement – ±1.96 * standard deviation mean difference, COV – coefficient of covariance, r –correlation coefficient, P – probability value, ICC – intra-class correlation coefficient, pAAo – proximal ascending aorta, dAAo – distal ascending aorta, AoA – Aortic arch, pDAo – proximal descending aorta and dDAo – distal descending aorta.

**Supplement 5 -** The inter-observer results of the clinical cohort per subgroup.

| Volume [cm^3^ = mL] | | | | | | | |
| --- | --- | --- | --- | --- | --- | --- | --- |
| Population | Analysis | Bland–Altman | | COV [%] | Pearson correlation | | ICC |
|  |  | Mean Diff | LoA |  | r | p |  |
| TAA | 1 | -1.5 | 8.5 | 11 | 0.97 | <0.01 | 0.97 |
|  | 2 | 0.5 | 17.8 | 24 | 0.79 | <0.01 | 0.88 |
|  | 3 | 2.0 | 16.0 | 21 | 0.78 | <0.01 | 0.87 |
| CoA | 1 | -1.1 | 1.5 | 10 | 0.97 | <0.01 | 0.96 |
|  | 2 | -0.7 | 2.0 | 13 | 0.95 | <0.01 | 0.96 |
|  | 3 | 0.4 | 2.5 | 16 | 0.92 | <0.01 | 0.95 |
| Healthy volunteers | 1 | -0.5 | 5.4 | 10 | 0.95 | <0.01 | 0.98 |
|  | 2 | 3.7 | 17.6 | 34 | 0.94 | <0.01 | 0.67 |
|  | 3 | 4.1 | 17.9 | 34 | 0.49 | <0.01 | 0.61 |
| Surface Area [cm^2^] | | | | | | | |
| Population | Analysis | Bland–Altman | | COV [%] | Pearson correlation | | ICC |
|  |  | Mean Diff | LoA |  | r | p |  |
| TAA | 1 | -2.3 | 8.3 | 9 | 0.95 | <0.01 | 0.94 |
|  | 2 | 0.6 | 16.2 | 17 | 0.67 | <0.01 | 0.79 |
|  | 3 | 2.9 | 13.1 | 14 | 0.67 | <0.01 | 0.78 |
| CoA | 1 | -1.6 | 2.5 | 8 | 0.96 | <0.01 | 0.95 |
|  | 2 | -1.3 | 3.0 | 9 | 0.94 | <0.01 | 0.95 |
|  | 3 | 0.3 | 4.1 | 12 | 0.89 | <0.01 | 0.94 |
| Healthy volunteers | 1 | -6.0 | 16.0 | 21 | 0.93 | <0.01 | 0.81 |
|  | 2 | -1.1 | 28.4 | 40 | 0.35 | 0.01 | 0.49 |
|  | 3 | 4.9 | 19.6 | 26 | 0.35 | 0.01 | 0.46 |
| Centerline Length [mm] | | | | | | | |
| Population | Seg | Bland–Altman | | COV [%] | Pearson correlation | | ICC |
|  |  | Mean Diff | LoA |  | r | p |  |
| TAA | 1 | -2.6 | 9.0 | 9 | 0.92 | <0.01 | 0.94 |
|  | 2 | 1.4 | 13.2 | 14 | 0.79 | <0.01 | 0.85 |
|  | 3 | 4.0 | 11.2 | 11 | 0.89 | <0.01 | 0.87 |
| CoA | 1 | -1.4 | 3.9 | 7 | 0.96 | <0.01 | 0.96 |
|  | 2 | -1.5 | 5.0 | 9 | 0.93 | <0.01 | 0.95 |
|  | 3 | -0.1 | 5.9 | 10 | 0.90 | <0.01 | 0.95 |
| Healthy volunteers | 1 | 2.2 | 9.4 | 10 | 0.86 | <0.01 | 0.90 |
|  | 2 | 6.2 | 14.3 | 16 | 0.67 | <0.01 | 0.70 |
|  | 3 | 4.0 | 15.0 | 17 | 0.54 | <0.01 | 0.65 |
| Maximal Diameter [mm] | | | | | | | |
| Population | Analysis | Bland-Altman | |  | Pearson correlation | | ICC |
|  |  | Mean Diff | LoA | COV [%] | r | p |  |
| TAA | 1 | -0.2 | 2.5 | 4 | 0.98 | <0.01 | 0.99 |
|  | 2 | 0.1 | 5.1 | 8 | 0.93 | <0.01 | 0.96 |
|  | 3 | 0.3 | 5.4 | 8 | 0.93 | <0.01 | 0.96 |
| CoA | 1 | -0.7 | 3.1 | 8 | 0.93 | <0.01 | 0.95 |
|  | 2 | -0.6 | 3.6 | 9 | 0.90 | <0.01 | 0.93 |
|  | 3 | 0.1 | 2.0 | 5 | 0.95 | <0.01 | 0.98 |
| Healthy volunteers | 1 | -0.9 | 1.2 | 2 | 0.99 | <0.01 | 0.98 |
|  | 2 | 0.4 | 7.8 | 14 | 0.63 | <0.01 | 0.76 |
|  | 3 | 1.3 | 8.0 | 14 | 0.62 | <0.01 | 0.75 |
| Curvature Radius [mm] | | | | | | | |
| Population | Analysis | Bland–Altman | | COV [%] | Pearson correlation | | ICC |
|  |  | Mean Diff | LoA |  | r | p |  |
| TAA | 1 | -1.5 | 16.7 | 14 | 0.97 | <0.01 | 0.98 |
|  | 2 | -0.9 | 37.5 | 31 | 0.85 | <0.01 | 0.92 |
|  | 3 | 0.6 | 0.6 | 32 | 0.83 | <0.01 | 0.91 |
| CoA | 1 | -5.3 | 43.6 | 43 | 0.87 | <0.01 | 0.89 |
|  | 2 | -5.4 | 26.3 | 26 | 0.92 | <0.01 | 0.95 |
|  | 3 | -0.2 | 46.6 | 44 | 0.83 | <0.01 | 0.90 |
| Healthy volunteers | 1 | 4.1 | 24.2 | 16 | 0.98 | <0.01 | 0.99 |
|  | 2 | 19.2 | 59.9 | 44 | 0.94 | <0.01 | 0.87 |
|  | 3 | 15.1 | 66.3 | 50 | 0.94 | <0.01 | 0.87 |

Results are presented per subgroup over all cardiac phases and anatomical segments (n=50). Abbreviations: Mean Diff –mean difference, Limits of agreement – ±1.96 * standard deviation mean difference, COV – coefficient of covariance, r –correlation coefficient, p – probability value, ICC – intra-class correlation coefficient.
